# Global mapping of the *Chlamydia trachomatis* conventional secreted effector – host interactome reveals CebN interacts with nucleoporins and Rae1 to impede STAT1 nuclear translocation

**DOI:** 10.1101/2024.04.25.587017

**Published:** 2024-04-25

**Authors:** Brianna Steiert, Shelby E. Andersen, Paige N. McCaslin, Cherilyn A. Elwell, Robert Faris, Xavier Tijerina, Parker Smith, Quinn Eldridge, Brian S. Imai, Justine V. Arrington, Peter M. Yau, Kathleen M. Mirrashidi, Jeffrey R. Johnson, Erik Verschueren, John Von Dollen, Gwendolyn M. Jang, Nevan J. Krogan, Joanne N. Engel, Mary M. Weber

**Affiliations:** 1 Department of Microbiology and Immunology, University of Iowa Carver College of Medicine, Iowa City, Iowa, USA.; 2 Present address: Department of Immunology and Microbiology, University of Colorado - Anschutz Medical Campus, Aurora, CO, USA; 3 Department of Medicine, University of California, San Francisco, San Francisco, CA, USA.; 4 Protein Sciences Facility, Roy J. Carver Biotechnology Center, University of Illinois Urbana–Champaign, Urbana, IL, USA; 5 QB3, California Institute for Quantitative Biosciences, San Francisco, CA 94148, USA; 6 Department of Cellular and Molecular Pharmacology, University of California, San Francisco, San Francisco, CA 94158, USA; 7 Gladstone Institutes, San Francisco, CA 94158, USA; 8 Department of Microbiology and Immunology, University of California, San Francisco, San Francisco, CA, USA.

**Keywords:** *Chlamydia trachomatis*, CT584, CebN, nucleoporin, Rae1, T3SS effector, Inc

## Abstract

*Chlamydia trachomatis* (*C.t*.), the leading cause of bacterial sexually transmitted infections, employs a type III secretion system (T3SS) to translocate two classes of effectors, inclusion membrane proteins and conventional T3SS (cT3SS) effectors, into the host cell to counter host defense mechanisms. Here we employed three assays to directly evaluate secretion during infection, validating secretion for 23 cT3SS effectors. As bioinformatic analyses have been largely unrevealing, we conducted affinity purification-mass spectrometry to identify host targets and gain insights into the functions of these effectors, identifying high confidence interacting partners for 21 cT3SS effectors. We demonstrate that CebN localizes to the nuclear envelope in infected and bystander cells where it interacts with multiple nucleoporins and Rae1, blocking STAT1 nuclear import following IFN-γ stimulation. By building a cT3SS effector-host interactome, we have identified novel pathways that are targeted during bacterial infection and have begun to address how *C.t.* effectors combat cell autonomous immunity.

## INTRODUCTION

To usurp host defense mechanisms and establish a favorable replicative niche, intracellular pathogens are tasked with remodeling the host cell using secreted virulence factors, termed effector proteins. Identification of the host proteins and pathways targeted by secreted proteins during active infections has been exceedingly difficult for obligate intracellular pathogens owing to their genetic intractability^1^. Several obligate intracellular pathogens, including *Chlamydia trachomatis* (*C.t.*), are the etiological agents of important human diseases for which no vaccines exist^2^. *C.t.* is the leading cause of infectious blindness and is the most common bacterial sexually transmitted infection worldwide^3^. Untreated infection can result in severe complications including pelvic inflammatory disease, ectopic pregnancy, sterility, and increased risk of developing cervical and ovarian cancer^4,5^. The incidence and prevalence of *C.t.* infections are rapidly rising, and reinfection rates are high due to a lack of long-term protective immunity and treatment failure following antibiotic therapy^2^. Understanding how *C.t.* co-opts the host cell to form its unique replicative niche is vital to developing new therapies.

All *Chlamydiae* share a biphasic developmental cycle in which the bacteria alternate between two forms: the infectious elementary body (EB) and the replicative reticulate body (RB)^3^. Upon contact with a target host cell, the EB delivers a set of pre-synthesized type III secretion system (T3SS) effector proteins into the eukaryotic cell to drive cytoskeletal rearrangements and membrane remodeling, triggering endocytosis of the pathogen^6–9^. The plasma membrane-derived compartment in which the EB resides is rapidly modified by the pathogen to form a unique replicative niche, termed the inclusion. The inclusion quickly dissociates from the endolysosomal pathway^10^, trafficking along microtubules to the peri-Golgi region^11^ where the EB differentiates into an RB and initiates replication. Following multiple rounds of division, RBs convert to EBs, and the bacteria are released by lysis or extrusion to begin the infection cycle anew^12^. While it is well established that formation of an intact replicative niche is vital for *C.t.* proliferation and chlamydial disease^13,14^, how *C.t.* accomplishes such feats remains incompletely understood.

Despite its extremely small genome (1Mb), *C.t.* is predicted to secrete an astonishing 10–15% of its proteome through the T3SS^1,15^. A large subset of these secreted proteins, termed inclusion membrane proteins (Incs), possess one or two bi-lobed hydrophobic domains of ~40 amino acids, allowing for intercalation into the inclusion membrane in such a way that the N- and C- termini of the protein are oriented into the host cell cytosol^16^. Inc proteins play a vital role at the host-pathogen interface; however, the other subset of effectors, conventional T3SS (cT3SS) effector proteins, are secreted into the host cell cytosol and likely also play vital roles in chlamydial infection at distal sites from the inclusion. Identification and functional characterization of cT3SS effector proteins has been challenging as most lack functional domains indicative of effector function, and they do not share homology to proteins with known functions. Until recently, *Chlamydiae* were recalcitrant to genetic manipulation, necessitating a reliance on surrogate systems to identify candidate secreted effectors^1^. To date, over 40 cT3SS effector proteins have been shown to be secreted in heterologous systems that employ *Yersinia pseudotuberculosis*, *Shigella flexneri*, or *Salmonella enterica* serovar Typhimurium as surrogate hosts^17–19^. While still challenging, advances in *Chlamydial* genetics, including the ability to transform *C.t.* with a stably maintained plasmid that enables inducible expression of epitope-tagged proteins and the adoption of multiple reporter constructs have allowed for validation of effector protein secretion during infection^20–22^. Significantly, several recent studies have confirmed that while useful, secretion by a surrogate organism does not necessarily correlate with secretion in the native organism^20,23^, necessitating validation of secretion by *C.t.*

Here we leveraged cutting-edge chlamydial genetics, in conjunction with large-scale unbiased affinity purification-mass spectrometry (AP-MS), to comprehensively define which cT3SS proteins are secreted by *C.t.* during infection and to map the host pathways targeted by these effector proteins. We identified 23 proteins that were secreted by *C.t.* in at least one assay and identified high-confidence interacting partners for 21 cT3SS proteins. Intriguingly, we show that CT584, which we have renamed *C**hlamydia* effector blocking nuclear transport (CebN), binds to a subset of Phe-Gly (FG) nucleoporins (NUPs) and the mRNA export factor Rae1 – targets previously associated with viral infection^24–28^. Our data indicate that CebN predominately localizes to the nuclear envelope in both infected and bystander cells and is sufficient to inhibit STAT1 import into the nucleus following interferon (IFN)-γ stimulation. This work significantly contributes to our understanding of *C.t.* cT3SS effectors and their host targets, providing a key stepping-stone for elucidating how these effector-host interactions contribute to the pathogenesis of *C.t.* infections.

## MATERIALS AND METHODS

### Bacterial and cell culture.

*Chlamydia trachomatis* serovar L2 (LGV 434/Bu) was propagated in HeLa 229 cells (American Type Culture Collection) and EBs were purified using a gastrografin density gradient as previously described^29^. HeLa cells were grown in RPMI 1640 medium (Thermo Fisher Scientific) supplemented with 10% Fetal Bovine Serum (Gibco) at 37°C with 5% CO_2_.

### Plasmid construction.

To assess secretion of candidate cT3SS effectors, chlamydial genes were PCR amplified from L2/434/Bu genomic DNA and each *orf* was cloned into the NotI/KpnI site of pBomb4 CyaA, pBomb4 BlaM, and pBomb4 GSK-FLAG^20^. For AP-MS, each validated cT3SS effector was cloned into the NotI/KpnI site of pBomb4-tet-mCherry with a FLAG-tag added to the C-terminus of each *orf* by PCR. CebN truncations were similarly cloned into pBomb4-tet-mCherry as FLAG-tagged fusions. For ectopic expression by transfection into human cells, secreted effectors were cloned into the KpnI/XhoI site of pcDNA3.1-GFP. The integrity of all constructs was verified by DNA sequencing at McLab. All primers used in this study are listed in Table S1.

### Transformation of *C.t.*

*C. trachomatis* serovar L2 (LGV 434/Bu) EBs were transformed as previously described^30^ with minor modifications. Briefly, plasmid DNA (5 µg), fresh *C.t.* lysates (~2 ×10^6^ EBs), and 10 µl 5X transformation mix (50 mM Tris pH 7.4 and 250 mM CaCl_2_) were gently mixed and the final volume was adjusted to 50 µl with tissue-culture grade water. Mixtures were incubated at room temperature for 30 min. RPMI with 10% FBS (4 ml) was then added to each transformation mix and 2 ml was applied to 2 wells of a 6-well plate containing a confluent HeLa cell monolayer. Plates were centrifuged at 900 x g for 30 min and at 18h post-infection, the media was replaced with RPMI with 10% FBS containing 0.3 µg/ml penicillin G (PenG). Infectious progeny were harvested every 48h and used for infections of a fresh HeLa cell monolayer until viable inclusions were evident (~2–3 passages). Expression of individual fusion proteins was confirmed by western blotting.

### Adenylate Cyclase (CyaA) secretion assay.

HeLa cell monolayers were infected at an MOI of 5 with *C.t.* transformants, and expression of the effector-CyaA fusion protein was induced using anhydrotetracycline hydrochloride (aTc; 10 ng/ml) as previously described^20^. cAMP production in host cells was quantified by ELISA according to the manufacturer’s guidelines (Abcam). Effector secretion was determined by comparing the levels of cAMP in cells infected with *C.t.* pBomb4 CyaA (negative control vector) to those infected with the *C.t.* CyaA-effector fusion strains.

### Beta-lactamase (BlaM) assay.

HeLa cells (2 × 10^4^/well) were seeded into black, clear bottom 96-well plates (Greiner). Cell monolayers were infected at an MOI of 5 and effector-β-lactamase (BlaM) fusion protein expression was induced using 10 ng/ml aTc as previously described^20^. At 24h post-infection (hpi), cells were washed three times with 1X PBS and loaded with CCF4-AM using the alternative loading protocol per the manufacturer’s instructions (ThermoFisher Scientific). Samples were incubated in the dark for 1h at room temperature and were then read on a Tecan Infinite M200 Pro plate reader. To quantify effector translocation, the background was subtracted, the ratio of 460nm to 535nm (blue:green) was determined, and expression relative to cells infected with *C.t.* pBomb4 BlaM (negative control vector) was calculated as previously described^31^.

### Glycogen synthase kinase (GSK)-FLAG immunoprecipitation.

Confluent HeLa cells monolayers in a T175 flask were infected at an MOI of 5 and expression of the effector-GSK-FLAG fusion was induced using 10 ng/ml aTc as previously described^20^. At 24 hpi, cells were washed with ice-cold 1X PBS, and lysed in 800 µl eukaryotic lysis solution (ELS) (50 mM Tris-HCl, 150 mM sodium chloride, 1 mM ethylenediaminetetraacetic acid, and 1% Triton-X 100) containing Halt cocktail protease and phosphatase inhibitor (ThermoFisher Scientific) along with 10 µM GSK-3 α and β inhibitor 1-(7-Methoxyquinolin-4-yl)-3-[6-(trifluoromethyl)pyridin-2-yl]urea (Tocris) as previously described^20^. Supernatants were applied to anti-FLAG magnetic beads (Pierce^™^ Thermo Fisher Scientific) for 1.5h at 4°C. The beads were subsequently washed 5X in ELS without Triton-X 100 and the purified protein was eluted using 4X NuPAGE LDS Sample Buffer (ThermoFisher Scientific). Samples were analyzed by western blotting.

### Western blotting.

For verification of CyaA or BlaM effector-fusion protein expression, confluent HeLa cell monolayers in a 6-well dish were infected at an MOI of 5. At 24 hpi, the samples were lysed in ELS with Halt cocktail protease inhibitor (ThermoFisher Scientific). For CyaA and BlaM, lysates were resolved using 3–8% Tris-Acetate protein gels with Tris-Acetate sodium dodecyl sulfate (SDS) running buffer. For GSK-FLAG IPs, samples were resolved using 4–12% Bis-Tris protein gels with (3-(N-morpholino)propanesulfonic acid) (MOPS)-SDS running buffer. Proteins were transferred to a PVDF membrane and probed using anti-CyaA (Santa Cruz), anti-BlaM (QED BioScience), anti-GSK-3β (Cell Signaling), or anti-Phospho-GSK-3β (Cell Signaling) antibodies using the concentrations listed in Table S2. For AP-MS expression verification and subsequent blots, samples were resolved either using 3–8% Tris-Acetate protein gels with Tris-Acetate SDS running buffer (proteins with MW >100kDa) or 4–12% Bis-Tris protein gels with MES running buffer (proteins with MW <100kDa). Proteins were transferred to a PVDF membrane and probed using anti-FLAG (Thermo Fisher Scientific), anti-GFP (Novus), anti-NUP54 (Proteintech, 16232-1-AP), anti-NUP153 (Novus, NBP1-81725), anti-NUP214 (abcam, AB70497) antibodies (Table S2).

### Immunofluorescence (IF) microscopy.

To determine the subcellular localization of secreted effector proteins, HeLa cells were transfected with pcDNA3.1-GFP plasmids using Lipofectamine LTX (Thermo Fisher Scientific). Cells were fixed with 4% formaldehyde 18h post-transfection and the nucleus was stained using DAPI (Invitrogen). Images were captured on a Nikon Ti2 immunofluorescent microscope.

For visualization of CebN by stimulated emission depletion (STED) microscopy, HeLa cells were transfected with pcDNA3.1-GFP plasmids containing an empty vector or CebN. Cells were fixed with 2% formaldehyde and permeabilized with 0.1% Triton-X 100 at 24h post-transfection and stained with DAPI and NUP specific antibodies: anti-NUP54, anti-NUP153, or anti-NUP214. Images were captured on a Leica SP8 inverted microscope. Images were deconvoluted using Imaris Professional Software.

For visualization of CebN during infection, HeLa cells were infected at an MOI of 2 with WT *C.t.* or *C.t.* strains expressing a FLAG-tagged empty vector, CebN-FLAG, or TmeA-FLAG. Expression was induced for 24h using 10 ng/ml aTc added at the time of infection. Cells were fixed with 4% formaldehyde 24h post-infection and stained with DAPI, anti-FLAG (Cell Signaling), and anti-*C.t.* HSP60 (Sigma). For STAT1 translocation experiments, HeLa cells were transfected with pcDNA3.1-GFP plasmids containing empty vector, CebN, or TmeA using Lipofectamine LTX (Thermo Fisher Scientific). Six h post transfection, the media was changed with half the samples receiving normal RPMI media and half with RPMI plus 600U/ml IFN-γ as previously described^32^. Cells were fixed with 4% formaldehyde 18h post-transfection and stained with DAPI and anti-STAT1 (Cell Signaling) antibody. Images were captured using a Leica DFC7000T confocal microscope equipped with Leica software. Nuclear translocation of STAT1 was quantified from 15 images per coverslip with three coverslips per biological replicate.

### Affinity Purification (AP):

HeLa cells, in three T175 flasks, were infected at an MOI of 2 with *C.t.* strains expressing a FLAG-tagged effector protein. Expression was induced for 24h using 10 ng/ml aTc, added at the time of infection. Four h prior to lysis, 10 µM MG132 (Millipore Sigma) was added to the media. Cells were subsequently lysed in ELS with Halt cocktail protease inhibitor. Lysates were centrifuged at 12,000 x g for 20 min, and the supernatants were incubated with 60 µl preclearing beads (mouse IgG agarose, Millipore Sigma) for 2h at 4°C. The precleared lysate was then incubated with 30 µl FLAG beads (anti-FLAG M2 Affinity Gel, Millipore Sigma) overnight at 4°C. The beads were washed six times with ELS without detergent. For mass spectrometry, samples were stored in 50 mM ammonium bicarbonate prior to digestion and analysis as previously described^33^. For western blotting, proteins were eluted from the beads in 4X NuPAGE LDS Sample Buffer (Thermo Fisher Scientific) and boiled for 5 minutes.

### Mass Spectrometry (MS).

MS was performed as previously described^33^, with the following adjustments. Beads containing samples were washed with 25 mM ammonium bicarbonate and digested with 0.5 micrograms trypsin (Pierce, Thermo Fisher Scientific, MS Grade) using a CEM microwave reactor for 30 min at 55°C. Digested peptides were extracted twice using 50% acetonitrile plus 5% formic acid, lyophilized to dryness, and resuspended in 5% acetonitrile plus 0.1% formic acid. For LC/MS, samples were injected into an UltiMate 3000 UHPLC system coupled online to a Thermo Scientific Orbitrap Fusion Tribrid mass spectrometer. Peptides were separated by reversed-phase chromatography using a 50-cm MicroPac Nano C18 column (Thermo Fisher Scientific) with mobile phases of 0.1% formic acid in water and 0.1% formic acid in acetonitrile; a linear gradient from 4% to 35% Acetonitrile over the course of 45 min was employed for peptide separations. The mass spectrometer was operated in a data-dependent acquisition (DDA) mode, employing precursor scans from 300 to 1,500 m/z (120,000 resolution) followed by collision induced dissociation (CID) of the most intense precursors over a maximum cycle time of 3 s (35% NCE, 1.6 m/z isolation window, 60-s dynamic exclusion window). Raw LC-MS/MS data were converted to peak lists using Mascot Distiller 2.8 and searched against a database containing UniProt_Human and Chlamydia_trachomatis_L2434Bu using Mascot 2.8 (Matrix Science). Tryptic digestion was specified with a maximum of two missed cleavages, while peptide and fragment mass tolerances were set to 10 ppm and 0.6 Daltons, respectively. Label-free Quantitation was performed utilizing the Mascot Average method on Mascot Distiller 2.8.2.

### Gene Ontology and pathway analysis.

Localization, Reactome pathway, biological process, and molecular function were determined for each host prey with a MiST^34^ score ≥0.69 using Uniprot and GeneCards. Dot plots for visualization were generated using R package ggplot2. STRING (12.0) was used to generate protein-protein interaction networks for each effector using those host prey with a MiST score ≥0.69. Cytoscape (3.10.1) was used for visualization of STRING networks.

### Statistics:

Statistical analysis was performed using GraphPad Prism 10.1.1 software. One-way ANOVAs were used followed by Tukey’s or Dunnett’s multiple comparisons with P < 0.05 (*), P < 0.01 (**), and P < 0.001 (***), P < 0.0001 (****).

## RESULTS

### Identification of cT3SS effector proteins that are secreted during *C.t.* infection.

Over 29 putative *C.t.* cT3SS effectors have been identified by their T3SS-dependent secretion in genetically tractable surrogate bacterial hosts^17–19,35,36^. Although useful, screens using surrogate hosts can yield false positives and negatives^20,23^. Thus, we applied newly developed *C.t.* genetics to directly test whether 29 candidate effectors (Table 1) are secreted into the host cell during *C.t.* infection. Additionally, we included several candidates from prior reports that were listed as negative for secretion due to incorrect product size during western blotting (CT016), low protein expression (CT309), or inconclusive results (CT330, CT338, CT386, CT504, and CT631)^36^. We also included TmeA and CteG in our assays as positive controls as they have been confirmed to be secreted by *C.t* ^21,22^. By applying 3 standard secretion assays that fuse the candidate protein of interest to an secretion dependent reporter construct, adenylate cyclase (CyaA), β-lactamase (BlaM), and GSK-FLAG (glycogen synthase kinase FLAG-tag)^20,21,23,37^, we sought to evaluate the secretion of 36 candidate secretion substrates during *C.t.* infection (Table 1). Prior to conducting the CyaA and BlaM reporter assays, expression of each candidate effector was confirmed by western blotting (Fig. S1). While we sought to evaluate secretion for all candidates in each of the 3 assays, some were not tested due to the inability to generate a clone, lack of *C.t.* transformants, or lack of expression (Table 1).

**Figure 1.**
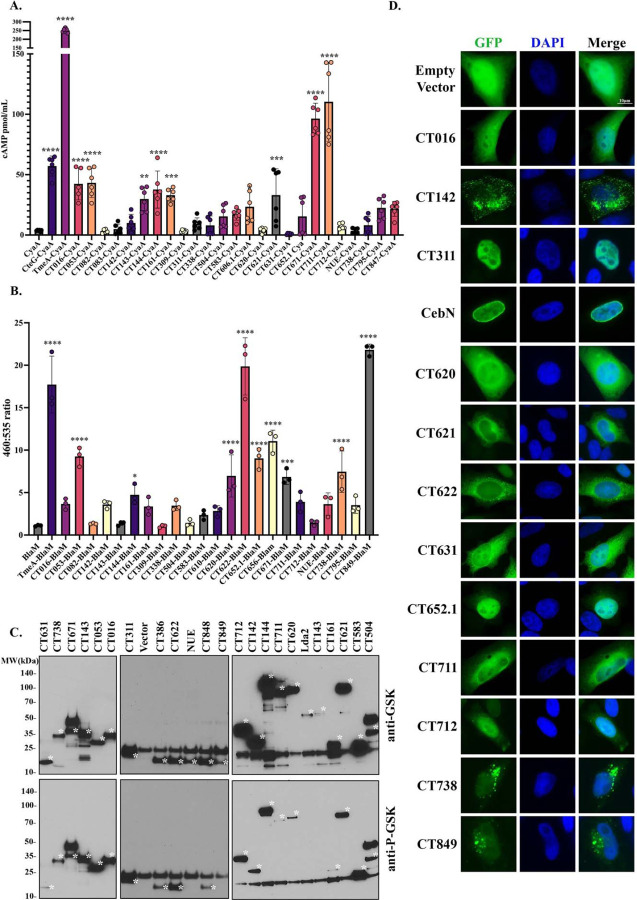
Identification of novel *C.t.* secreted proteins using CyaA, BlaM, and GSK secretion assays and preliminary characterization by ectopic expression. Candidate secretion substrates were expressed as C-terminal fusions to the CyaA, BlaM, or GSK-FLAG tags and transformed into *C.t.* Transformants were used to infect HeLa cells at an MOI of 5 for 24h. After 24h, (A) cytosolic levels of cAMP were measured by competitive ELISA and compared to CyaA alone. (B) Infected cells were loaded with CCF4-AM for 1h and the change in 460/535nm fluorescence was monitored on a plate reader. Ratios associated with the candidate substrate-BlaM fusions were compared to *C.t.* expressing BlaM alone. (C) The candidate peptide was immunoprecipitated using anti-FLAG beads. Samples were resolved by SDS-PAGE and immunoblots were probed with anti-GSK or anti-P-GSK to evaluate expression and secretion, respectively. Asterisk indicates predicted molecular weight of each GSK-FLAG fusion. (A, B) Statistical significance was determined using One-Way ANOVA with Dunnett’s as a post-test comparing to CyaA or BlaM alone. ****P<0.0001, ***P<0.001, **P<0.01, *P<0.001. (D) cT3SS effectors confirmed to be secreted were expressed as N-terminal GFP fusions (green). The host nucleus was stained with DAPI (blue). Images were collected for each effector and compared to the pattern of GFP alone. (A-D) Data are representative of three independent experiments.

Using TmeA-CyaA and CteG-CyaA as positive controls, we demonstrate that infection of HeLa cells with these *C.t.* strains yield significantly elevated intracellular levels of cAMP relative to cells infected with *C.t.* expressing CyaA alone (Fig. 1A). These results confirm that secretion of *C.t.* cT3SS effectors can readily be detected using this approach and can be applied to identify other secreted factors. Applying this approach to the candidate secretion substrates, we show that CT016, CT053, CT143, CT144, CT161, CT606.1, CT621, CT671, and CT711 are capable of being translocated into the host cell during *C.t.* infection (Fig.1A, Table 1). To evaluate candidate effector secretion using a second assay, we fused BlaM to the C-terminus of each candidate and confirmed the functionality of this assay using TmeA-BlaM as a positive control (Fig.1B), which was previously shown to be secreted using this assay^21^. Here we demonstrate that CT053, CT144, CT620, CT622, CT652.1, CT656, CT671, CT738, and CT849 are secreted by *C.t.* using the BlaM assay (Fig.1B, Table 1). Lastly, we employed the GSK assay to evaluate effector secretion as this assay has the advantage of using a small, 13-residue tag that can be phosphorylated in the host cell cytosol. Using this approach, we demonstrate that CT016, CT053, CT142, CT143, CT144, CT161, CT311, CT386, CT504, CT583, CT620, CT621, CT622, CT631, CT671, CT711, CT712, CT738, and CT848 are secreted (Fig.1C, Table 1). The GSK-fusion protein assay appeared to be the most sensitive, likely owing to the smaller size of the tag allowing for more efficient translocation. For subsequent analysis, we required that the effectors be secreted in at least 2 assays to be classified as “secreted,” in one assay to be identified as “possibly secreted,” and “not secreted” if negative in at least 2 assays and not positive in any assay. Collectively, of the 36 candidates tested, we identified 11 secreted and 12 possibly secreted effectors (Table 1).

### Subcellular localization of transfected cT3SS effector-GFP fusion proteins.

Following translocation into a host cell, many effector proteins localize to specific subcellular compartments where they interact with host proteins^38–41^. To identify host organelles targeted by the 11 secreted and 12 possibly secreted effectors, as well as CebN (which we have previously shown to be secreted^20^), we used IF microscopy. Transient transfection of HeLa cells with GFP-fusion proteins revealed that 12/23 cT3SS effectors displayed a pattern distinct from GFP alone (Fig 1D). CT311, CebN, and CT652.1 localized to the nucleus, with CebN being enriched at the nuclear envelope. CT620, CT621, CT622, CT631, CT711, and CT712 were excluded from the nucleus and displayed a cytosolic pattern. CT142, CT738, and CT849 also localized to the cytoplasm, but accumulated in punctate-like structures (Fig. 1D).

### Identification of putative host proteins and pathways targeted by cT3SS effector proteins during *C.t.* infection.

A few cT3SS effector proteins have been functionally characterized and shown to modulate diverse host cell signaling pathways^6,8,9,19,33,42–44^. However, the function of most of these putative virulence factors remains unknown. Affinity purification-mass spectrometry (AP-MS) has emerged as a powerful technique to comprehensively map protein-protein interactions (PPIs) between bacterial effectors or viral proteins and host proteins, yielding key mechanistic insights into how these pathogens establish their unique replicative niches^34,45–48^. While informative, most of these studies have been undertaken by overexpressing a single effector protein in a mammalian cell at non-physiological levels and in the absence of additional bacterial or viral factors that might promote or hinder PPIs. With the increasing genetic tractability of *C.t.*, we are poised to evaluate effector-host PPIs in the context of infection.

Here we sought to perform AP-MS on a total of 33 cT3SS effectors: 23 from this study, 9 additional recently identified effectors^20^, and TmeB^21^. Of these, 24 were successfully expressed in *C.t.* and the remaining effector proteins were excluded from further analysis due to the inability to obtain chlamydial transformants or due to the lack of detectable expression by western blotting. Only prey proteins with at least 2 unique peptides that were present in at least 2 of the replicates were further pursued. CT311 and CT161 were excluded from further analysis due to lack of detection of the bait protein following AP-MS. To identify high-confidence PPIs, we analyzed the complete data set using Mass Spectrometry interaction STatistics (MiST) (Table S3), which evaluates prey reproducibility, abundance, and specificity to generate scores between 0 and 1^34^. Using a cut-off score of ≥ 0.69, we identified 241 putative host interacting partners for 21 cT3SS effectors. While CT144 was detected in the AP-MS, and multiple preys were identified, none of these were predicted high-confidence interactions when scored by MiST.

**Figure 2.**
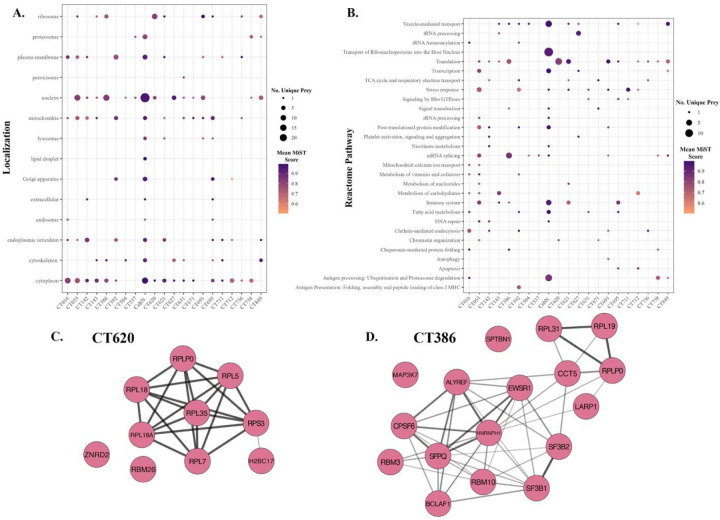
Host pathways identified as targets of *C.t.* secreted effector proteins using AP-MS. (A,B) MiST was used to identify high confidence interacting partners for each effector screened. Those with a MiST score ≥0.69 were considered significant and used for further analysis of (A) subcellular localization and (B) Reactome pathways targeted by each effector using UniProt and Genecards. Size of the dot correlates with the number of unique prey(s) and color correlates with average MiST score for those prey. (C, D) STRING and Cytoscape were used to generate STRING network maps to evaluate potential connections between prey. CT620 (C) and CT386 (D) showed strong networks of interconnecting prey proteins identified by AP-MS. Darker, thicker lines correlate with more known and predicted interactions between node proteins.

To further define the potential function of the *C.t.* cT3SS effector proteins, Gene Ontology (Genecards) and pathway analysis (Reactome and Uniprot) was performed for each of the 241 MiST high-confidence interactors (Table S3) (Fig. 2A, B). As shown in Fig. 2A, most of the host proteins targeted by *C.t.* effectors reside in the cytoplasm, nucleus, endoplasmic reticulum, and mitochondria; however, a few host proteins associated with the Golgi apparatus, ribosomes, cytoskeleton, and plasma membrane were also noted. Pathway analysis revealed that these *C.t.* cT3SS effector proteins target host protein associated with vesicle-mediated transport, translation, stress response, mRNA splicing, and the immune system (Fig. 2B). In agreement with our pathway analysis, the majority of the MiST high-confidence interactors function in protein or RNA binding (Fig. S2A) to facilitate protein transport, transcription, or translation (Fig. S2B).

**Figure 3.**
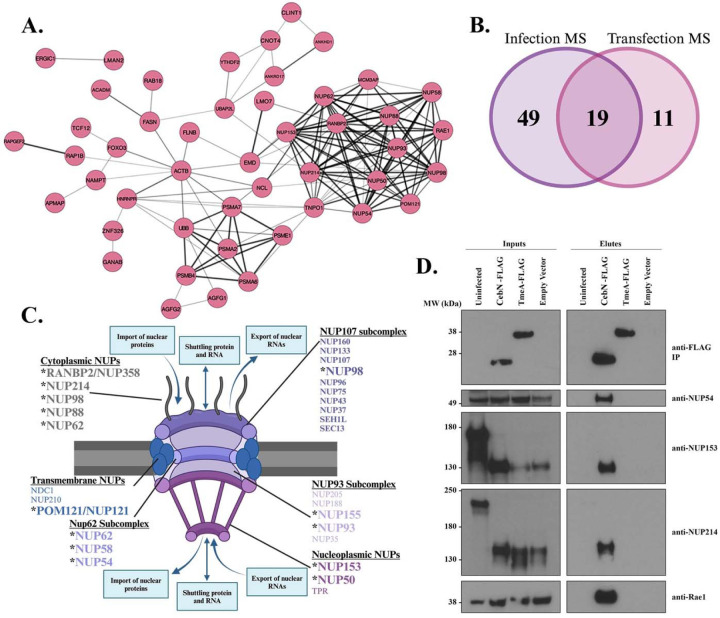
CebN interacts with multiple nucleoporins and Rae1. (A) STRING and Cytoscape were used to generate a network map for CebN, which revealed an enrichment for nucleoporin (NUP) proteins. Darker, thicker lines correlate with more known and predicted interactions between node proteins. (B) Transfection AP-MS was used to complement the infection AP-MS and showed a strong overlap of identified host targets. (C) Schematic of the nuclear pore complex. Bolded and * NUPs were identified as putative CebN targets using AP-MS. (D) IP of *C.t.* expressing FLAG-tagged vector, CebN, or TmeA from HeLa cells. Blots were probed with specific NUP and Rae1 antibodies. Data are representative of three replicates.

To further delineate potential effector function, we employed STRING and Cytoscape to map PPI networks and identify effector proteins that associated with multiprotein complexes (Fig. 2C, D, 3A, and S3). Through assessment of whether prey proteins that were pulled down by individual effector proteins interacted with one another we show that the cT3SS effector proteins CT620, CT386, and CebN associated with multiprotein complexes (Fig. 2C, D, 3A). Notably strong connections between proteins that are structural components of the ribosome and are important for translation were noted for CT620 (Fig. 2C), suggesting that this effector might target the translation machinery (Fig. 2B). Analysis of the predicted high confidence host protein interactors of CT386 identified a subset of RNA-binding proteins associated with mRNA processing (Fig. 2A, D), suggesting CT386 might also interfere with translation.

### Ectopically expressed CebN binds to multiple nucleoporins and Rae1.

Our infection AP-MS screen identified 68 high-confidence interactors for CebN (Table S3). Using STRING and Cytoscape, we assembled a PPI network map for CebN, revealing that many of its targets are involved in transport of ribonucleoproteins into the nucleus, mRNA export, or transcription (Fig. 3A). Transfected CebN-GFP predominately localized to the nuclear envelope (Fig. 1D), a pattern that aligns with it binding to host proteins involved in nucleocytoplasmic transport. To confirm these interactions, and to rule out the requirement of additional bacterial proteins that contribute to the CebN infection interactome, we performed AP-MS on Strep-tagged CebN as previously described^48^. This approach identified 30 high-confidence interactors (MIST ≥ 0.69) for CebN (Table S3), of which 19 overlapped with the infection IP (Table 2, Fig. 3B). Notably, 9 nucleoporins (NUP58, NUP214, NUP98, NUP54, NUP62, NUP88, NUP153, POM121/NUP121 and RANBP2/NUP358) and the mRNA export factor Rae1 were present in both the CebN infection and transfection interactomes.

Nucleoporins (NUPs) are a family of ~30 proteins that form the nuclear pore complex (NPC) and play an important role in regulating import and export of small molecules into and out of the nucleus^49^. The NPC is organized into an inner pore ring, the nuclear and cytoplasmic rings, the nuclear basket, and the cytoplasmic filaments, each of which are enriched for select NUPs (Fig. 3C)^49^. Intriguingly, while most of the NUPs that CebN binds make up the cytoplasmic filaments, interactions with NUPs in other subcomplexes of the NPC were noted (Fig. 3C), suggesting that secreted CebN may play a broad role in modulating NPC function. Rae1 is an mRNA export factor that binds to NUP98 to aid in the transport of messenger ribonucleoprotein (mRNP) complexes through the nuclear pore complex^50^. Several viral proteins target NUPs and Rae1 to promote replication of their genomic information and to dampen the host response to infection by blocking import of important transcription factors^25–28^. To the best of our knowledge, no bacterial protein has been identified that targets NUP proteins or Rae1, making CebN an intriguing effector protein to study.

### CebN binds to and co-localizes with NUPs and Rae1.

To confirm CebN binding to NUP proteins and Rae1, we immunoprecipitated FLAG tagged CebN from *C.t.* infected cells and probed with antibodies specific to NUPs and Rae1. We focused on NUP54, NUP153, and NUP214 due to their high peptide counts in the AP-MS (Table S3). NUP54, NUP153, NUP214, and Rae1 IP with CebN but not with vector or TmeA (Fig. 3D), an effector previously shown to bind to N-WASP^6,8^. Processing of NUP153 and NUP214 was noted on these blots in infected samples. We determined that CPAF, a broad-spectrum protease produced by *C.t.* was responsible for this post-lysis cleavage, as this processing was absent in lysates derived from HeLa cells infected with a CPAF mutant^51^ (Fig. S4).

**Figure 4.**
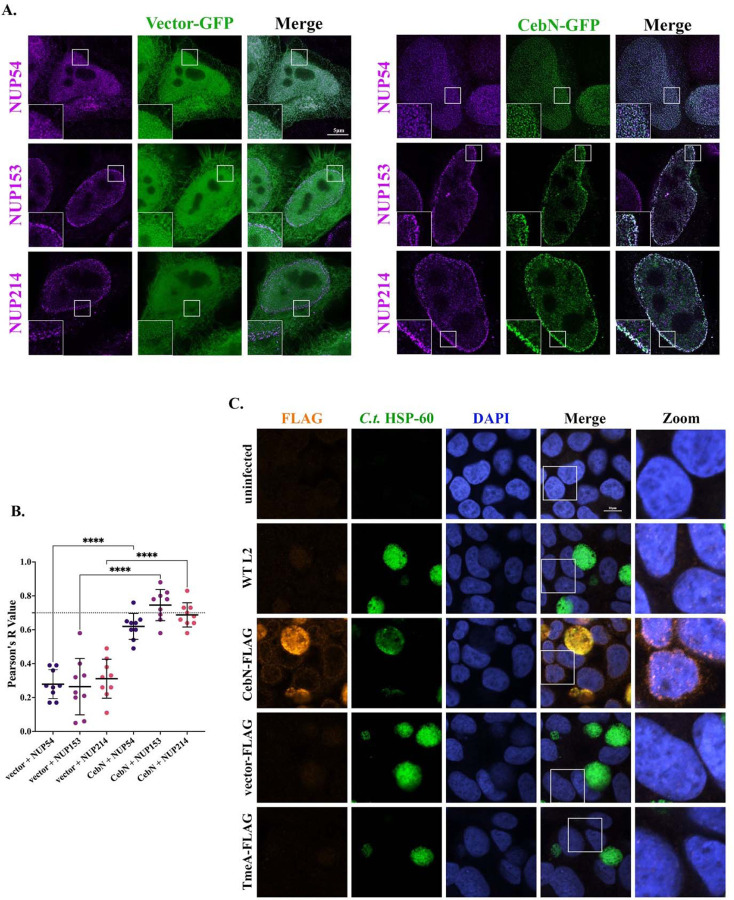
CebN localizes to the nuclear envelope in transfection and infection conditions. (A) STED images of HeLa cells transfected with GFP-tagged empty vector or GFP-CebN (green) and stained with NUP specific antibodies (magenta). (B) Quantification of colocalization (white) was determined using Fiji and Pearson’s correlation coefficient with significance shown between GFP-vector and GFP-CebN transfected samples. Graph displays individual values, mean (black line), and standard deviation. ****P<0.0001 Significance was determined using one-way ANOVA followed by Tukey’s multiple comparisons test. (C) HeLa cells uninfected or infected with WT L2 or *C.t.* expressing FLAG-tagged vector, CebN, or TmeA (negative control). Cells were fixed with 4% formaldehyde and stained with FLAG (orange), *C.t.* HSP-60 (green) and DAPI (blue). Zoomed image depicts overexposed nuclear envelopes of infected and/or bystander cells. (A, C) Data are representative of three replicates.

To additionally confirm the interaction between CebN and NUPs, HeLa cells were transfected with GFP-CebN or GFP-vector, fixed, and stained using anti-NUP54, NUP153, and NUP214 antibodies. Imaging by stimulated emission depletion (STED) microscopy confirmed that CebN colocalized (white) with specific NUP proteins, whereas no colocalization was noted with GFP-vector (Fig. 4A, B). Pearson’s correlation coefficient was calculated as a measure of colocalization, and a significant difference was found between GFP-vector and GFP-CebN transfected cells for each individual NUP (Fig. 4B). Taken together our results indicate that the cT3SS effector protein CebN localizes to the nuclear envelope where it binds to multiple NUPs and to Rae1.

### CebN localizes to the nuclear envelope of infected and bystander cells.

Most cT3SS effector proteins are not readily visualized by microscopy, and thus their subcellular localization is generally assessed by transfection. Due to CebN’s unique localization to the nuclear envelope, we assessed CebN localization directly in *C.t.* infected cells. In line with our ectopic expression data, CebN-FLAG was found to localize to the nuclear envelope of infected cells (Fig. 4C). Intriguingly, we also observed CebN on the nuclear envelopes of bystander cells (Fig. 4C), suggesting this effector can translocate into adjacent cells.

### The C-terminus of CebN is required for interactions with NUPs and Rae1 as well as for its localization to nuclear envelope.

**Figure 5.**
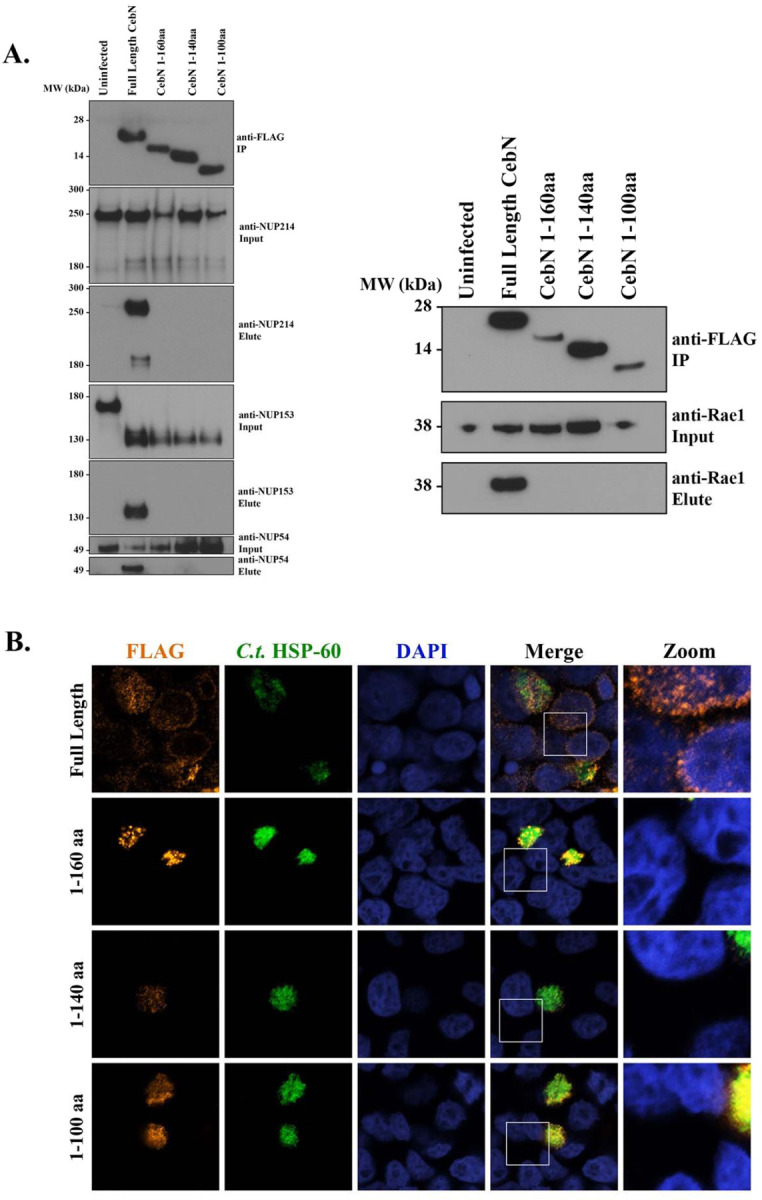
The C-terminus of CebN is necessary for interaction with NUPs and Rae1 as well as for localization to the nuclear envelope. (A) Lysates from HeLa cells infected for 24 h with *C.t.* expressing the indicated C-terminally FLAG-tagged CebN constructs were IP’d on FLAG beads and immunoblotted with the indicated antibodies. (B) HeLa cells infected for 24 h with *C.t.* expressing the indicated C-terminally FLAG-tagged CebN constructs were fixed with 4% formaldehyde and stained with antibodies to FLAG (orange), *C.t.* HSP-60 (green, to visualize bacteria) and with DAPI (blue). Zoomed image depicts overexposed nuclear envelopes of infected and/or bystander cells. (A-B) Data are representative of at least two replicates.

To delineate the region of CebN that is necessary for interaction with NUPs and Rae1, we generated 20–40 amino acid sequential truncations from the C-terminus of the 183 amino acid protein and expressed these truncations as FLAG-tagged constructs in *C.t.* Immunoprecipitation of these truncations, followed by subsequent western blotting, showed that the C-terminal 23 amino acids of CebN are necessary for interactions with NUP54, NUP153, NUP214, and Rae1 (Fig. 5A). We further confirmed the importance of this region by performing confocal microscopy on HeLa cells infected with the *C.t.* strains expressing the CebN FLAG-tagged deletion constructs (Fig 5B). As noted above, full length CebN-FLAG localizes to the nuclear envelope of infected cells, as well as the nuclear envelopes of bystander cells (Fig. 5B). However, none of the truncated versions of CebN localized to the nuclear envelope in infected or bystander cells (Fig. 5B). In total, the C-terminus of CebN is necessary for its interaction with NUPs and Rae1 and for its localization to the nuclear envelope in infected and bystander cells.

### CebN attenuates STAT1 import into the nucleus following interferon-γ stimulation.

**Figure 6.**
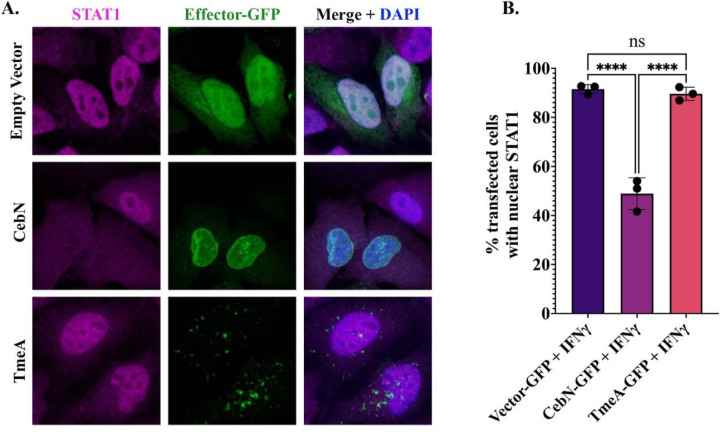
Ectopically expressed CebN is sufficient to block IFN-γ-stimulated STAT1 nuclear translocation. (A) Cells were transfected with GFP-tagged vector, GFP-CebN, or GFP-TmeA (green) and treated with 600U/ml IFN-γ. Fixed cells were stained with anti-STAT1 (magenta) antibody and DAPI to demark the nucleus (blue). (B) Translocation of STAT1 into the nucleus was quantified from 15 images per coverslips with three coverslips per biological replicate. Error bars indicate SD. ****P<0.0001. Significance was determined using one-way ANOVA followed by Tukey’s multiple comparisons test. (A, B) Data are representative of three replicates.

Viral proteins from HIV, SARS-CoV-2, Kaposi’s sarcoma-associated herpesvirus, and vesicular stomatitis virus interact with and rearrange the nuclear pore complex to modulate nuclear import of transcription factors required for the anti-viral response^25,26,28,52–58^. Similarly, *C.t.* attenuates nuclear import of STAT1 following IFN-γ stimulation^32^. We hypothesized that CebN interactions with NUPs and Rae1 could be responsible for perturbing STAT1 nuclear import. To test this, HeLa cells were transfected with GFP-CebN, GFP-empty vector, or GFP-TmeA, treated with IFN-γ, and imaged by confocal IF microscopy using an anti-STAT1 antibody (Fig. 6). We observed a significant decrease in the frequency of cells with nuclear translocated STAT1 between CebN-transfected cells compared to those cells transfected with empty vector or TmeA. Thus, CebN by itself is sufficient to block the translocation of STAT1 into the nucleus. Taken together, our results suggest that CebN, through interactions with nucleoporins and Rae1, plays a key role in dampening the host response to *C.t.* infection by blocking nuclear translocation of a key transcriptional regulator of the host innate immune response, STAT1.

## DISCUSSION

In this study, we combined newly developed *C.t.* genetics with AP-MS to definitively identify *C.t.* cT3SS effectors and to generate the first cT3SS effector-host interactome. Our approach successfully identified high confidence interacting host partners for 21 of the 36 uncharacterized cT3SS effectors. Our work is especially valuable as it not only begins to build a compendium of proteins that are secreted into the host cell during active infection, but also provides a launch point for detailed mechanistic characterization of these effector proteins. Importantly, screens such as AP-MS, can reveal novel pathways targeted by intracellular bacteria^45–47^. Here we discovered that CebN targets NUPs to inhibit STAT1 nuclear import, revealing a potential mechanism by which *C.t.* neutralizes the host innate immune response to survive intracellularly. Altogether, these studies will give us a better understanding of how obligate intracellular pathogens remodel the host to form their unique replicative niches.

One of the most striking findings from our AP-MS screen was the interaction between CebN and multiple nucleoporins and Rae1. Pointing to its importance in *C.t.* intracellular infection, CebN is 100% conserved amongst *C.t.* serovars, including trachoma (serovars A-C), urogenital (serovars D-K), and lymphogranuloma venereum (L1-L3) isolates and is highly conserved (83% identical and 91% similar) in *C. pneumoniae*^59^. Previous biophysical studies on CebN were interpreted as this protein associating with the tip of the T3SS needle apparatus^59^, whereas studies using *Yersinia enterocolitica* as a surrogate host indicated CebN might function as a chaperone^35^. Subsequent protein-protein interaction studies indicated that CebN interacts with core components of the T3SS, including the needle protein CdsF, the ATPase CdsN, and the C-ring CdsF^60,61^, suggesting it might be a T3SS effector protein. In agreement with this observation, we have shown that CebN is secreted into the host cell during *C.t.* infection^20^ and in the current study, we begin to mechanistically dissect the role of CebN in co-opting NUP and Rae1 functions.

While our study is, to the best of our knowledge, the first time a bacterial effector has been shown to interact with host nucleoporin proteins, several viral proteins have been identified that co-opt NUPs and Rae1. ORF6 of SARS-CoV-2, ORF10 of Kaposi’s sarcoma-associated herpesvirus, and M protein of vesicular stomatitis virus all bind to the NUP98-Rae1 complex, whereas the HIV-1 capsid binds to multiple nucleoporins leading to altered NUP expression and localization^25,26,28,52–58^. While HIV-1 manipulates NUPs to facilitate viral import and integration of its genome into the host genome^62^, other viral proteins interact with NUPs to alter nucleocytoplasmic transport of key transcription factors like STAT1. During SARS-CoV-2 infection, ORF6-Rae1-NUP98 interactions block STAT1 import into the nucleus and mRNA export, resulting in a significantly diminished host response^24,26,28,57^. The ability of CebN to perturb STAT1 import during *C.t.* infection and our observation that it binds to NUPs-Rae1 suggest it might perturb host defense mechanisms by an analogous mechanism to that of ORF6 during SARS-CoV-2 infection.

Similar to how viruses dampen the immune response, *C.t.* has been shown to antagonize interferon pathways^63^. *C.t.* infection induces production of the proinflammatory cytokine IFN-γ, which is aimed at curtailing the infection, however the bacteria can attenuate this response establishing a persistent infection^64^. IFN production by the host cell activates the JAK-STAT signaling pathway. Activation leads to phosphorylation and homodimerization of STAT1, which is imported into the nucleus by karyopherin alpha 1 and karyopherin beta 1 heterodimers^49^. Once in the nucleus, the STAT1 homodimer complex binds to gamma-activated site promoter elements to drive expression of a subset of ISGs meant to impede the infection. Recent studies have shown that following IFN-γ stimulation, nuclear translocation of STAT1 is reduced^32^ and inhibition of the JAK-STAT pathway correlates with lower mRNA and protein levels of key interferon response elements in *C.t.* infected cells compared to uninfected cells^65^. Furthermore, this difference was dependent on *C.t. de novo* protein synthesis, supporting the role for a *C.t.* effector protein in this process^65^. Our new data showing that CebN binds NUPs and Rae1 and perturbs nuclear translocation of STAT1 may be the missing link needed to understand how *C.t.* establishes a persistent infection in spite of robust IFN-γ production. While the exact mechanism for how CebN-NUP-Rae1 interactions lead to blocked STAT1 nuclear translocation and whether this leads to diminished ISG production remains to be elucidated, our new observations support a role for CebN in attenuation of the IFN-γ response during *C.t.* infection. Future studies employing a CebN deletion mutant may yield additional information about its role in antagonizing the host defense system in the context of infection. However, as *C.t.* is predicted to use multiple mechanisms to antagonize the host immune response, our CebN transfection studies highlight the importance of this individual effector protein to this process.

Lining the NPC are intrinsically disordered NUPs that harbor numerous Phe-Gly (FG) repeats separated by a hydrophilic spacer of 5–30 amino acids^49^. Movement of large cargo across the NPC requires highly specific interactions between these so-called FG-NUPs and transporters of the karyopherin family, which enables entry and rapid diffusion of the cargo-transporter complex through the NPC. Of the 11 NUPs identified as putative binding partners of CebN, 9 are classified as FG-NUPs (Table 2, S3). Recognition of FG motifs within these select NUPs might explain how CebN binds to multiple nucleoporins.

Crystallization of CebN revealed that the N-terminus consists of a four α-helix bundles (α1-α2-α3-α4), followed by a three-stranded antiparallel β-sheets (β1-β2-β3)^66^. The C-terminus contains two α-helices (α5 Val135-Lys154 and α6 Pro156-Leu179) arranged in a kinked antiparallel manner^66^. AlphaFold modeling predicts that the C-terminus of CebN harbors a coiled-coil domain (amino acids 153–181). Truncation of the last 23 amino acids of CebN would disrupt this predicted coiled-coil, thus abrogating binding to NUPs and Rae1. While analysis of CebN did not identify motifs known to be required for interactions with NUPs or Rae1, new motifs are constantly being discovered, and it is possible that CebN possesses a previously undefined motif.

Ectopically expressed CebN appeared to concentrate at the nuclear envelope, thus we sought to evaluate the localization of CebN during infection. Upon doing so, not only did we observe CebN at the nuclear envelope of infected cells, but we also observed localization to the nuclear envelope of bystander cells. This unique localization in apparently uninfected bystander cells has only ever observed once before with the *C. psittaci* cT3SS effector SINC, which similarly targets the nuclear envelope through interactions with lamins^67^. Two mechanisms for SINC exit from infected cells into presumably uninfected bystander cells were proposed: packaging within exosomes for release at the cell surface and tunneling nanotubes. Prior work with *Mycobacterium tuberculosis* has revealed that mycobacterial proteins are packaged into vesicles and released via calcium-regulated lysosomal exocytosis. These proteins are then trafficked to uninfected bystander cells, expanding the microorganism’s sphere of influence to other cells without the need to infect them^68^. Tunneling nanotubes are transient cellular connections that play a role in cell-to-cell communication and facilitate exchange of molecules between cells. Analogous to viruses, previous work has shown that *C.t.* may spread cell-to-cell by nanotubules^69^. Thus, it is conceivable that effector proteins may also be transmitted to adjacent cells via this mechanism. If an infected cell undergoes cell division, the inclusion is partitioned into one cell, leaving the other daughter cell “uninfected^70^.” One intriguing possibility is that secreted effector proteins are left behind and continue to function in the absence of infection. The ability of *C.t.* effectors to access neighboring cells has huge implications for infection, potentially allowing *C.t.* to prime neighboring cells for infection prior to the invasion of the bacterium. Future work will involve delineating the exact mechanism by which *C.t.* is able to transport effector proteins into neighboring, uninfected cells and further whether effectors besides CebN and SINC can access adjacent cells.

Of the 36 effectors we sought to test for secretion in *C.t.*, 23 were found to be secreted in at least one assay. Due to challenges generating clones, obtaining chlamydial transformants, and/or lack of expression during *C.t.* infection, we were unable to draw conclusions for 8 of these candidates. Of note, CT082, NUE, and CT795 were found to be not secreted in our assays (Table 1). NUE was previously shown to be secreted using *Shigella flexneri* as a surrogate host and mechanistic characterization revealed it localizes to the nucleus and interacts with chromatin^42^. While screening in a surrogate host is useful, especially in genetically intractable organisms, we^20^ and others^23^ have shown that secretion by a surrogate organism does not necessarily correlate with secretion in the native host. Our new results suggest NUE might not be secreted by *C.t.* However, it is possible that localization to the nucleus hinders evaluation of secretion and that assays such as subcellular fractionation of infected cells could tell whether it truly localizes in the nucleus.

As with all screens, false positives and negatives can result. To add rigor to our AP-MS data set analysis, we employed MiST, which combines metrics of reproducibility, specificity, and abundance across the entire data set to identify putative host binding partners more accurately and stringently. Using this technique, we identified high confidence targets for 21 of the secreted effectors tested herein. In our study, we sought to define PPIs for all the previously uncharacterized cT3SS effector proteins and included CT695 (TmeB) as at the onset of this study TmeB had no function ascribed to it. Recent work has since shown that ectopically expressed TmeB targets the ARP2/3 complex^71^. While we did not find components of the ARP2/3 complex in our infection AP-MS, we did identify an actin-binding protein, inverted formin 2 (INF2). INF2 belongs to the formin family of proteins, which function to both polymerize and depolymerize actin filaments^72^. Formins and the ARP2/3 complex act in parallel to regulate the actin cytoskeleton^73^. Differences in experimental set-up between transfection of TmeB-FLAG and infection of a *C.t.* strain expressing TmeB-FLAG could contribute to these differences in identified putative host binding partners. Additionally, our timepoint of 24 hours post infection may correlate with functions of TmeB beyond invasion.

**Figure 7.**
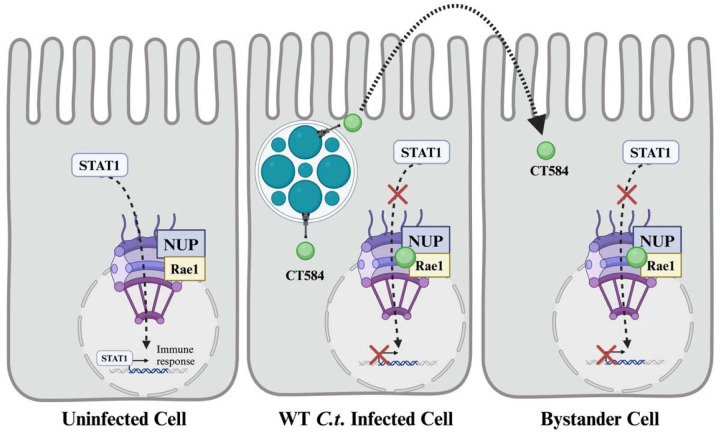
Schematic of working model. In uninfected cells, IFN-γ production triggers STAT1 translocation to the nucleus through the nuclear pore complex where it binds to gamma-activated site (GAS) promoter elements to drive expression of a subset of ISGs meant to impede the infection. In *C.t.* infected cells, STAT1 translocation is blocked by CebN, which interacts with NUPs and Rae1, ultimately dampening the transcriptional regulation potential of STAT1. Additionally, CebN is translocated to neighboring cells, potentially priming these cells for infection through altered nuclear import and/or export.

By combining chlamydial genetics with large-scale screens, we have begun to define the compendium of proteins secreted during active *C.t.* infection and further have identified putative host proteins and pathways targeted by each of these important factors. Our analysis uncovered a wealth of unique high-confidence host interactors, which will lay the groundwork for detailed mechanistic characterization of cT3SS effector proteins. Importantly, our approach has identified targets previously not associated with bacterial infection, including nucleoporins, which are commonly targeted by viral proteins to dampen the host response to viral infection^25–28^ . We propose a model whereby secretion of CebN leads to inhibition of STAT1 nuclear translocation through interaction of CebN with NUPs and Rae1 (Fig. 7), which we hypothesize ultimately alters host cell transcription to downregulate the innate immune response. More research is needed to know exactly what genes are being regulated. Moreover, the ability of CebN to translocate to bystander cells may provide a mechanism by which *C.t.* can prime nearby cells for infection. Future study of how CebN co-opts NUPs and Rae1 will not only enhance our understanding of how *C.t.* establishes a persistent infection despite a robust host response but may also identify druggable targets applicable to both bacterial and viral infections.

## Supplementary Material

Supplement 1

## Figures and Tables

**Table 1:** Candidate cT3SS effectors evaluated for secretion in *C.t.* ND- Not Determined

D/UW-3/CX	L2/434/Bu	Reference	MW	CyaA Assay	BlaM Assay	GSK Assay	Final Designation
CT016	CTL0271	^36^	26.7kDa	Secreted	Not Secreted	Secreted	Secreted
CT053	CTL0309	^36^	17.2kDa	Secreted	Secreted	Secreted	Secreted
CT082	CTL0338	^35,36,74^	60kDa	Not Secreted	Not Secreted	Not Secreted	Not Secreted
CT083	CTL0338A	^18^	18.3kDa	Not Secreted	ND^b^	ND^c^	Unable to conclude
CT142	CTL0397	^36,75^	31.4kDa	Not Secreted	Not Secreted	Secreted	Possibly Secreted
CT143	CTL0398	^36,75^	31.5kDa	Secreted	Not Secreted	Secreted	Secreted
CT144	CTL0399	^36,75^	31.4kDa	Secreted	Secreted	Secreted	Secreted
CT161	CTL0417	^36^	28.0kDa	Secreted	Not Secreted	Secreted	Secreted
CT163	CTL0419	^76^	59.1kDa	ND^b^	ND^d^	Not Secreted	Unable to conclude
CT309	CTL0561	^36^	32.1kDa	Not Secreted	Not Secreted	ND^d^	Not Secreted
CT311	CTL0563	^77^	26.3kDa	Not Secreted	ND^c^	Secreted	Possibly Secreted
CT330	CTL0584	^36^	10.0kDa	ND^b^	ND^d^	ND^d^	Unable to conclude
CT338	CTL0592	^36^	17.8kDa	Not Secreted	Not Secreted	ND^d^	Not Secreted
CT386	CTL0642	^36^	33.1kDa	ND^c^	ND^a^	Secreted	Possibly Secreted
CT429	CTL0688	^36^	39.2kDa	ND^c^	ND^c^	ND^d^	Unable to conclude
CT504	CTL0766	^36^	32.0kDa	Not Secreted	Not Secreted	Secreted	Possibly Secreted
CT550	CTL0812	^18^	15.8kDa	ND^a^	ND^c^	ND^b^	Unable to conclude
CT583	CTL0846	^36^	30.7kDa	Not Secreted	Not Secreted	Secreted	Possibly Secreted
CT606.1	CTL0870	^18^	8.9kDa	Secreted	ND^c^	ND^c^	Possibly Secreted
CT610	CTL0874	^18^	26.8kDa	ND^b^	Not Secreted	ND^c^	Unable to conclude
CT620	CTL0884	^17^	93.2kDa	Not Secreted	Secreted	Secreted	Secreted
CT621	CTL0885	^17^	92.6kDa	Secreted	ND^b^	Secreted	Secreted
CT622	CTL0886	^78^	21.7kDa	ND^a^	Secreted	Secreted	Secreted
CT631	CTL0895	^36^	9.2kDa	Not Secreted	ND^b^	Secreted	Possibly Secreted
CT652.1	CTL0021	^18^	6.6kDa	Not Secreted	Secreted	ND^a^	Possibly Secreted
CT656	CTL0025	^36^	11.2kDa	ND^c^	Secreted	ND^c^	Possibly Secreted
CT671	CTL0040	^18^	31.0kDa	Secreted	Secreted	Secreted	Secreted
CT711	CTL0080	^17^	86.3kDa	Secreted	Not Secreted	Secreted	Secreted
CT712	CTL0081	^17^	44.1kDa	Not Secreted	Not Secreted	Secreted	Possibly Secreted
CT718	CTL0087	^18^	19.6kDa	ND^c^	ND^c^	ND^c^	Unable to conclude
CT737 (NUE)	CTL0106	^42^	25.7kDa	Not Secreted	Not Secreted	Not Secreted	Not Secreted
CT738	CTL0107	^18^	29.1kDa	Not Secreted	Secreted	Secreted	Secreted
CT795	CTL0164	^79^	17.9kDa	Not Secreted	Not Secreted	ND^b^	Not Secreted
CT847	CTL0219	^19^	18.9kDa	Not Secreted	ND^b^	ND^b^	Unable to conclude
CT848	CTL0220	^18^	18.3kDa	ND^c^	ND^b^	Secreted	Possibly Secreted
CT849	CTL0221	^36^	17.5kDa	ND^b^	Secreted	Not Secreted	Possibly Secreted

a unable to generate clone

b unable to obtain chlamydial transformants

c not expressed

d skipped, positive already in two.

**Table 2:** Host proteins with significant MiST score (>0.69) common to the infection and transfection IPs of CebN.

Gene	Subcellular Localization	Molecular Function	Biological Process	Transfection IP MiST	Infection IP MiST
HNRNPR	nucleoplasm	RNA binding	mRNA processing	1.000	0.992
NUP58	nuclear pore	structural component of nuclear pore complex	transport: nucleocytoplasmic	0.995	0.993
NUP214	nuclear pore	structural component of nuclear pore complex	transport: nucleocytoplasmic	0.994	0.994
NUP98	nuclear pore	structural component of nuclear pore complex	transport: nucleocytoplasmic	0.993	0.994
RAE1	nuclear pore	RNA binding	transport: nucleocytoplasmic	0.991	0.992
TCF12	nucleosome	DNA binding	transcription	0.990	0.993
CNOT4	cytoplasm	transferase activity	ubiquitin-dependent protein catabolic process	0.989	0.986
NUP54	nuclear pore	structural component of nuclear pore complex	transport: nucleocytoplasmic	0.986	0.989
ZNF326	nuclear matrix	RNA binding	mRNA processing	0.982	0.986
NUP62	nuclear pore	structural component of nuclear pore complex	transport: nucleocytoplasmic	0.973	0.993
NUP88	nuclear pore	structural component of nuclear pore complex	transport: nucleocytoplasmic	0.972	0.992
POM121	nuclear pore	structural component of nuclear pore complex	transport: nucleocytoplasmic	0.971	0.968
AGFG1	nuclear pore	GTPase activator activity	mRNA export	0.963	0.988
NUP153	nuclear pore	structural component of nuclear pore complex	transport: nucleocytoplasmic	0.961	0.994
UNK	cytoplasm	RNA binding	translation	0.946	0.988
ANKRD17	nucleoplasm	protein binding	immune system process	0.911	0.983
TMEM131	membrane	unknown	unknown	0.866	0.994
ANKHD1	cytoplasm	protein binding	immune system process	0.826	0.983
RANBP2	nuclear pore	ligase activity	transport: nucleocytoplasmic	0.690	0.966
