# Supplementary material for: Global mapping of the Chlamydia trachomatis conventional secreted effector – host interactome reveals CebN interacts with nucleoporins and Rae1 to impede STAT1 nuclear translocation
Source: bioRxiv. 2024 Apr 25:2024.04.25.587017. Preprint. [Version 1] doi: 10.1101/2024.04.25.587017 (PMC11071493; doi:10.1101/2024.04.25.587017)
Supplement: Supplementary file 1 [file media-1.zip › Sup/TableS1 Primer FINAL.docx]

Table S1: Primers used for completion of this study. Nucleotides in bold correspond to the restriction site. Nucleotides in blue correspond to the FLAG-tag. IP = constructs used for immunoprecipitation, IF = constructs used for immunofluorescence

| **Primer Name** | **Sequence** | **Uses** |
| --- | --- | --- |
| **Expression in** ***C.t.*** |  |  |
| CT016 NotI F | CC**GCGGCCGC**ATGAAAGTCAAAATTAATGATCAGTTC | CyaA, BlaM, GSK assays and AP-MS |
| CT016 KpnI R | CC**GGTACC**AGTATAAAGAACAGCTTTCACGTGTTC | CyaA, BlaM, GSK assays |
| CT016 FLAG KpnI R | CC**GGTACCTTACTTATCGTCGTCATCCTTGTAATC**AGTATAAAGAACAGCTTTCACGTGTTC | AP-MS |
| CT053 NotI F | CC**GCGGCCGC**ATGAAAAGTGAGCGTTTAAAAAAATT | CyaA, BlaM, GSK assays and AP-MS |
| CT053 KpnI R | CC**GGTACC**CCATTCATTCGCGTCAGG | CyaA, BlaM, GSK assays |
| CT053 FLAG KpnI R | CC**GGTACCTTACTTATCGTCGTCATCCTTGTAATC**CCATTCATTCGCGTCAGG | AP-MS |
| CT082 NotI F | CC**GCGGCCGC**ATGTCAATTTCTGGAAGTGGTAATG | CyaA, BlaM, GSK assays |
| CT082 KpnI R | CC**GGTACC**TGAATCGCCGCCTGC | CyaA, BlaM, GSK assays |
| CT083 NotI F | CC**GCGGCCGC**ATGAGAATAATCCCTTTTGATCCTT | CyaA, BlaM, GSK assays |
| CT083 KpnI R | CC**GGTACC**TGAATCGCCGCCTGC | CyaA, BlaM, GSK assays |
| CT142 NotI F | CC**GCGGCCGC**ATGAGTGATTCTGACAAAATTATTAAT | CyaA, BlaM, GSK assays and AP-MS |
| CT142 KpnI R | CC**GGTACC**TCCTCCTATCTCTGGGTATACGAG | CyaA, BlaM, GSK assays |
| CT142 FLAG KpnI R | CC**GGTACCTTACTTATCGTCGTCATCCTTGTAATC**TCCTCCTATCTCTGGGTATACGAG | AP-MS |
| CT143 NotI F | CC**GCGGCCGC**ATGAAGAAACCAGTATTTACAGGGG | CyaA, BlaM, GSK assays and AP-MS |
| CT143 KpnI R | CC**GGTACC**ATCTGCCTCCTTATAAGAAGAACCA | CyaA, BlaM, GSK assays |
| CT143 FLAG KpnI R | CC**GGTACCTTACTTATCGTCGTCATCCTTGTAATC**ATCTGCCTCCTTATAAGAAGAACCA | AP-MS |
| CT144 NotI F | CC**GCGGCCGC**ATGACAACACCAGATAATAATACTATTGAT | CyaA, BlaM, GSK assays and AP-MS |
| CT144 KpnI R | CC**GGTACC**AGGAACAACAGGTAGCCGAA | CyaA, BlaM, GSK assays |
| CT144 FLAG KpnI R | CC**GGTACCTTACTTATCGTCGTCATCCTTGTAATC**AGGAACAACAGGTAGCCGAA | AP-MS |
| CT161 NotI F | CC**GCGGCCGC**GTGGCTAGAAAACCTTTAGTAGATAGA | CyaA, BlaM, GSK assays and AP-MS |
| CT161 KpnI R | CC**GGTACC**GTCATAAAAATTTTCCATTTCTGTAGG | CyaA, BlaM, GSK assays |
| CT161 FLAG KpnI R | CC**GGTACCTTACTTATCGTCGTCATCCTTGTAATC**GTCATAAAAATTTTCCATTTCTGTAGG | AP-MS |
| CT163 NotI F | CC**GCGGCCGC**ATGTTTGTGTCGTTCGATAAATCC | CyaA, BlaM, GSK assays |
| CT163 KpnI R | CC**GGTACC**ATAACGAATGCGACAATACTGC | CyaA, BlaM, GSK assays |
| CT309 NotI F | CC**GCGGCCGC**ATGAATCAGTATTATTTTTTATCCTCCTTC | CyaA, BlaM, GSK assays |
| CT309 KpnI R | CC**GGTACC**CCATCTGATTCCTTTCTCCAT | CyaA, BlaM, GSK assays |
| CT311 NotI F | CC**GCGGCCGC**ATGAAAAGAGTTATCCTCTGCTCTCT | CyaA, BlaM, GSK assays |
| CT311 KpnI R | CC**GGTACC**TTTTCCATTTTGCAGATCTTTCA | CyaA, BlaM, GSK assays |
| CT311 FLAG KpnI R | CC**GGTACCTTACTTATCGTCGTCATCCTTGTAATC**TTTTCCATTTTGCAGATCTTTCA | AP-MS |
| CT330 NotI F | CC**GCGGCCGC**ATGCAGGAAATCTCGGTACCT | CyaA, BlaM, GSK assays |
| CT330 KpnI R | CC**GGTACC**TACAGATTCCCCAGGGATAAAAG | CyaA, BlaM, GSK assays |
| CT338 NotI F | CC**GCGGCCGC**ATGGTGTATAGTTATAAAGGAATAGTATATTT | CyaA, BlaM, GSK assays |
| CT338 KpnI R | CC**GGTACC**AGGTTTTTGAGATAAAAGATACT | CyaA, BlaM, GSK assays |
| CT386 NotI F | CC**GCGGCCGC**ATGCAAATTCCAAGAAGTGTTG | CyaA, BlaM, GSK assays |
| CT386 KpnI R | CC**GGTACC**TACTAATCTCTGCTGTTTTAACA | CyaA, BlaM, GSK assays |
| CT386 FLAG KpnI R | CC**GGTACCTTACTTATCGTCGTCATCCTTGTAATC**TACTAATCTCTGCTGTTTTAACA | AP-MS |
| CT392 NotI F | CC**GCGGCCGC**ATGTCTTCTATACAAGGAAC | AP-MS |
| CT392 FLAG KpnI R | CC**GGTACCTTACTTATCGTCGTCATCCTTGTAATC**AAATCCTCTATCATCATCGG | AP-MS |
| CT429 NotI F | CC**GCGGCCGC**ATGACGACATATCCTGTACCTCAA | CyaA, BlaM, GSK assays |
| CT429 KpnI R | CC**GGTACC**TGAACGGCTCTTCTTACGTCCAC | CyaA, BlaM, GSK assays |
| CT504 NotI F | CC**GCGGCCGC**GTGTATTTTACAAGAGATCCAGTCAT | CyaA, BlaM, GSK assays |
| CT504 KpnI R | CC**GGTACC**CTCTTCTGAAGAAATACTGTC | CyaA, BlaM, GSK assays |
| CT504 FLAG KpnI R | CC**GGTACCTTACTTATCGTCGTCATCCTTGTAATC**CTCTTCTGAAGAAATACTGTC | AP-MS |
| CT537 NotI F | CC**GCGGCCGC**ATGGGTAGATACAGAAGAG | AP-MS |
| CT537 FLAG KpnI R | CC**GGTACC**ATCGTTCTCCATAAAAAAGC | AP-MS |
| CT550 NotI F | CC**GCGGCCGC**GTGAGTTTAGATTTTTTAGAGGA | CyaA, BlaM, GSK assays |
| CT550 KpnI R | CC**GGTACC**TCCATCTATTGAAGGCG | CyaA, BlaM, GSK assays |
| CT583 NotI F | CC**GCGGCCGC**ATGGGAAATATTAAAACCCTTTTAGAG | CyaA, BlaM, GSK assays |
| CT583 KpnI R | CC**GGTACC**TCGATTTCTAGAGTTTTGGGTTT | CyaA, BlaM, GSK assays |
| CT583 FLAG KpnI R | CC**GGTACCTTACTTATCGTCGTCATCCTTGTAATC**TCGATTTCTAGAGTTTTGGGTTT | AP-MS |
| CT584 NotI F | CC**GCGGCCGC**ATGACGACGAAACCCAAAACTCT | AP-MS, IP, IF |
| CT584 FLAG KpnI R | CC**GGTACCTTACTTATCGTCGTCATCCTTGTAATC**CACAGATTTCGTTAATTCTTCAA | AP-MS, IP, IF |
| CT584 1-160aa FLAG KpnI R | CC**GGTACCTTACTTATCGTCGTCATCCTTGTAATC**TTTTTCTCCGATAGGATTTTT | IP, IF |
| CT584 1-140aa FLAG KpnI R | CC**GGTACCTTACTTATCGTCGTCATCCTTGTAATC**ATGGCAGGTATTTTGTACATCCG | IP, IF |
| CT584 1-100aa FLAG KpnI R | CC**GGTACCTTACTTATCGTCGTCATCCTTGTAATC**GAATTCACCGCGCTCATGG | IP, IF |
| CT606.1 NotI F | CC**GCGGCCGC**TTGGAAGATAGAATGATCGACGG | CyaA, BlaM, GSK assays |
| CT606.1 KpnI R | CC**GGTACC**CTCGCGGGGAAAGAGAGTCT | CyaA, BlaM, GSK assays |
| CT606.1 FLAG KpnI R | CC**GGTACCTTACTTATCGTCGTCATCCTTGTAATC**CTCGCGGGGAAAGAGAGTCT | AP-MS |
| CT610 NotI F | CC**GCGGCCGC**ATGATGGAGGTGTTTATGAAT | CyaA, BlaM, GSK assays |
| CT610 KpnI R | CC**GGTACC**ATAAGATTGATGACAACTACAAC | CyaA, BlaM, GSK assays |
| CT610 FLAG KpnI R | CC**GGTACCTTACTTATCGTCGTCATCCTTGTAATC**ATAAGATTGATGACAACTACAAC | AP-MS |
| CT620 NotI F | CC**GCGGCCGC**ATGTGTTCTATGAACATATTTAATAAAATTAACTC | CyaA, BlaM, GSK assays |
| CT620 KpnI R | CC**GGTACC**ACTAGCCAGTTTTCTTGTTAAACCA | CyaA, BlaM, GSK assays |
| CT620 FLAG SalI R | CC**GTCGACTTACTTATCGTCGTCATCCTTGTAATC**ACTAGCCAGTTTTCTTGTTAAACCA | AP-MS |
| CT621 NotI F | CC**GCGGCCGC**ATGAACCGTATTCATCGTACACAA | CyaA, BlaM, GSK assays |
| CT621 KpnI R | CC**GGTACC**TCTTAAGAGATTACGCGCTAATCC | CyaA, BlaM, GSK assays |
| CT621 FLAG SalI R | CC**GTCGACTTACTTATCGTCGTCATCCTTGTAATC**TCTTAAGAGATTACGCGCTAATCC | AP-MS |
| CT622 NotI F | CC**GCGGCCGC**ATGGAATCAGGACCAGAATCAG | CyaA, BlaM, GSK assays |
| CT622 KpnI R | CC**GGTACC**AGAAAGATAACCAGAGAATAGAGAA | CyaA, BlaM, GSK assays |
| CT622 FLAG SalI R | CC**GTCGACTTACTTATCGTCGTCATCCTTGTAATC**AGAAAGATAACCAGAGAATAGAGAA | AP-MS |
| CT627 NotI F | CC**GCGGCCGC**ATGGAAAAGAATTATTATGC | AP-MS |
| CT627 FLAG KpnI R | CC**GGTACCTTACTTATCGTCGTCATCCTTGTAATC**TGCTGGTTGGTTTTCTTGTTCTT | AP-MS |
| CT631 NotI F | CC**GCGGCCGC**ATGAAAACGTTAATTGATAACA | CyaA, BlaM, GSK assays |
| CT631 KpnI R | CC**GGTACC**TAAACAAATAATTCCTTCAAACT | CyaA, BlaM, GSK assays |
| CT631 FLAG KpnI R | CC**GGTACCTTACTTATCGTCGTCATCCTTGTAATC**TAAACAAATAATTCCTTCAAACT | AP-MS |
| CT652.1 NotI F | CC**GCGGCCGC**ATGGACCAGTTATCACAGA | CyaA, BlaM, GSK assays |
| CT652.1 KpnI R | CC**GGTACC**ACCTTGGGAATCTTCTT | CyaA, BlaM, GSK assays |
| CT652.1 FLAG KpnI R | CC**GGTACCTTACTTATCGTCGTCATCCTTGTAATC**ACCTTGGGAATCTTCTT | AP-MS |
| CT656 NotI F | CC**GCGGCCGC**ATGGACACGCAATTCATAGCG | CyaA, BlaM, GSK assays |
| CT656 KpnI R | CC**GGTACC**ATCTCTGTATACCGAACGCATTT | CyaA, BlaM, GSK assays |
| CT656 FLAG KpnI R | CC**GGTACCTTACTTATCGTCGTCATCCTTGTAATC**ATCTCTGTATACCGAACGCATTT | AP-MS |
| CT671 NotI F | CC**GCGGCCGC**ATGGAATTAAATAAAACTTCGGAATCT | CyaA, BlaM, GSK assays |
| CT671 KpnI R | CC**GGTACC**TATATGAGCTTCTTCTACTTTCTTCTC | CyaA, BlaM, GSK assays |
| CT671 FLAG KpnI R | CC**GTCGACTTACTTATCGTCGTCATCCTTGTAATC**TATATGAGCTTCTTCTACTTTCTTCTC | AP-MS |
| CT691 NotI F | CC**GCGGCCGC**ATGCAAGTCCTAGCAAGTTTATT | AP-MS |
| CT691 FLAG KpnI R | CC**GTCGACTTACTTATCGTCGTCATCCTTGTAATC**TTTCTCTTCTAGCGTCATACTCA | AP-MS |
| CT694 (TmeA) NotI F | CC**GCGGCCGC**ATGAGTATTCGACCTACTAATGGGAG | IP, IF |
| CT694 (TmeA) FLAG SalI | CC**GTCGACTTACTTATCGTCGTCATCCTTGTAATC**GTCTAAGAAAACAGAAGAAGTTATGAC | IP, IF |
| CT695 NotI F | CC**GCGGCCGC**GTGAGTAGCATAAGCCCTATAGGG | AP-MS |
| CT695 FLAG KpnI R | CC**GTCGACTTACTTATCGTCGTCATCCTTGTAATC**GATATTCCCAACCGAAGAAGG | AP-MS |
| CT711 NotI F | CC**GCGGCCGC**GTGTCAATACAACCTACATCCATTTC | CyaA, BlaM, GSK assays |
| CT711 KpnI R | CC**GGTACC**TTTAAATCTACGGATCAACTTAGCAA | CyaA, BlaM, GSK assays |
| CT711 FLAG KpnI R | CC**GGTACCTTACTTATCGTCGTCATCCTTGTAATC**TTTAAATCTACGGATCAACTTAGCAA | AP-MS |
| CT712 NotI F | CC**GCGGCCGC**ATGAGAAACCATCCGATTCC | CyaA, BlaM, GSK assays |
| CT712 KpnI R | CC**GGTACC**GCTAGAAGCCAATGTTCTATATACATT | CyaA, BlaM, GSK assays |
| CT712 FLAG KpnI R | CC**GGTACCTTACTTATCGTCGTCATCCTTGTAATC**GCTAGAAGCCAATGTTCTATATACATT | AP-MS |
| CT718 NotI F | CC**GCGGCCGC**TTGGAGGATTACGTGGCTTC | CyaA, BlaM, GSK assays |
| CT718 KpnI R | CC**GGTACC**TGGAGTGAGTACACTTAGTAGATGGG | CyaA, BlaM, GSK assays |
| CT736 NotI F | CC**GCGGCCGC**ATGCAACTCACCTCACAAG | AP-MS |
| CT736 FLAG KpnI R | CC**GTCGACTTACTTATCGTCGTCATCCTTGTAATC**GTCTTTTTCATATGTGCCC | AP-MS |
| CT737 NotI F | CC**GCGGCCGC**ATGACCACTAACTCTACTCAAGACACT | CyaA, BlaM, GSK assays |
| CT737 KpnI R | CC**GGTACC**TTCTTCTTGGGGAACGAAT | CyaA, BlaM, GSK assays |
| CT738 NotI F | CC**GCGGCCGC**ATGGAAGGTTTTTTCCCTATA | CyaA, BlaM, GSK assays |
| CT738 KpnI R | CC**GGTACC**TACAAGGCTCGGAGCAGAAAAGA | CyaA, BlaM, GSK assays |
| CT738 FLAG SalI R | CC**GGTACCTTACTTATCGTCGTCATCCTTGTAATC**TACAAGGCTCGGAGCAGAAAAGA | AP-MS |
| CT795 NotI F | CC**GCGGCCGC**ATGAGATTCTTGTTAGCTTTATTCTCA | CyaA, BlaM, GSK assays |
| CT795 KpnI R | CC**GGTACC**CTCAACAAATTCAGGATTTATTG | CyaA, BlaM, GSK assays |
| CT847 NotI F | CC**GCGGCCGC**ATGAGTAGTGCAATCATCCCC | CyaA, BlaM, GSK assays |
| CT847 KpnI R | CC**GGTACC**GTCCGAAGGAGACGTTGTAAG | CyaA, BlaM, GSK assays |
| CT848 NotI F | CC**GCGGCCGC**ATGTGGCATAAAGAACCAATGTATG | CyaA, BlaM, GSK assays |
| CT848 KpnI R | CC**GGTACC**GATATTCGCGATCAAGCTAACG | CyaA, BlaM, GSK assays |
| CT848 FLAG SalI R | CC**GTCGACTTACTTATCGTCGTCATCCTTGTAATC**GATATTCGCGATCAAGCTAACG | AP-MS |
| CT849 NotI F | CC**GCGGCCGC**ATGTCAGCACCAACCTCACA | CyaA, BlaM, GSK assays |
| CT849 KpnI R | CC**GGTACC**AGACAGGGGTTTATTTAATTGGTTAAC | CyaA, BlaM, GSK assays |
| CT849 FLAG SalI R | CC**GTCGACTTACTTATCGTCGTCATCCTTGTAATC**AGACAGGGGTTTATTTAATTGGTTAAC | AP-MS |
| **Ectopic Expression** |  |  |
| CT016 KpnI F | CC**GGTACC**ATGAAAGTCAAAATTAATGATCAGTTC | Ectopic expression |
| CT016 XhoI R | CC**CTCGAG**AGTATAAAGAACAGCTTTCACGTGTTC | Ectopic expression |
| CT053 KpnI F | CC**GGTACC**ATGAAAAGTGAGCGTTTAA | Ectopic expression |
| CT053 XhoI R | CC**CTCGAG**CCATTCATTCGCGTCAGG | Ectopic expression |
| CT142 KpnI F | CC**GGTACC**ATGAGTGATTCTGACAAAATTATTAAT | Ectopic expression |
| CT142 XbaI R | CC**TCTAGA**TTATCCTCCTATCTCTGGGTATACGAG | Ectopic expression |
| CT143 KpnI F | CC**GGTACC**TTAATCTGCCTCCTTATAAGAAGAACCA | Ectopic expression |
| CT143 XhoI R | CC**CTCGAG**ATCTGCCTCCTTATAAGAAGAACCA | Ectopic expression |
| CT144 KpnI F | CC**GGTACC**ATGACAACACCAGATAATAATACTATTGAT | Ectopic expression |
| CT144 XhoI R | CC**CTCGAG**AGGAACAACAGGTAGCCGAA | Ectopic expression |
| CT161 KpnI F | CC**GGTACC**GTGGCTAGAAAACCTTTAGTAGATAGA | Ectopic expression |
| CT161 XhoI R | CC**CTCGAG**GTCATAAAAATTTTCCATTTCTGTAGG | Ectopic expression |
| CT311 KpnI F | CC**GGTACC**ATGAAAAGAGTTATCCTCTGCTCTCT | Ectopic expression |
| CT311 XhoI R | CC**CTCGAG**TTTTCCATTTTGCAGATCTTTCA | Ectopic expression |
| CT386 KpnI F | CC**GGTACC**ATGCAAATTCCAAGAAGTGTTG | Ectopic expression |
| CT386 XhoI R | CC**GGTACC**TACTAATCTCTGCTGTTTTAACA | Ectopic expression |
| CT504 KpnI F | CC**CTCGAG**GTGTATTTTACAAGAGATCCAGTCAT | Ectopic expression |
| CT504 XhoI R | CC**GGTACC**CTCTTCTGAAGAAATACTGTC | Ectopic expression |
| CT583 KpnI F | CC**GGTACC**ATGGGAAATATTAAAACCCTTTTAGAG | Ectopic expression |
| CT583 EcoRI R | CC**GAATTC**TCGATTTCTAGAGTTTTGGGTTT | Ectopic expression |
| CT584 KpnI F | CC**GGTACC**ATGACGACGAAACCCAAAACTCT | Ectopic expression |
| CT584 SalI R | CC**GTCGAC**TTACACAGATTTCGTTAATTCTT | Ectopic expression |
| CT620 KpnI F | CC**GGTACC**ATGTGTTCTATGAACATATTTAATAAAATTAACTC | Ectopic expression |
| CT620 XhoI R | CC**CTCGAG**ACTAGCCAGTTTTCTTGTTAAACCA | Ectopic expression |
| CT621 KpnI F | CC**GGTACC**ATGAACCGTATTCATCGTACACAA | Ectopic expression |
| CT621 EcoRI R | CC**GAATTC**TCTTAAGAGATTACGCGCTAATCC | Ectopic expression |
| CT622 KpnI F | CC**GGTACC**ATGGAATCAGGACCAGAATCAG | Ectopic expression |
| CT622 XhoI R | CC**CTCGAG**AGAAAGATAACCAGAGAATAGAGAAGC | Ectopic expression |
| CT631 KpnI F | CCGGTACCATGAAAACGTTAATTGATAACAACATC | Ectopic expression |
| CT631 XhoI R | CC**CTCGAG**TAAACAAATAATTCCTTCAAACT | Ectopic expression |
| CT652.1 KpnI F | CC**GGTACC**ATGGACCAGTTATCACAGATACATCA | Ectopic expression |
| CT651.2 XhoI R | CC**CTCGAGTTA**ACCTTGGGAATCTTCTTCGG | Ectopic expression |
| CT656 KpnI F | CCGGTACCATGGACACGCAATTCATAGC | Ectopic expression |
| CT656 XhoI R | CC**CTCGAGTTA**ATCTCTGTATACCGAACGCATTTT | Ectopic expression |
| CT671 KpnI F | CC**GGTACC**ATGGAATTAAATAAAACTTCGGAATCT | Ectopic expression |
| CT671 XhoI R | CC**CTCGAGTTA**TATATGAGCTTCTTCTACTTTCTTCTC | Ectopic expression |
| CT694 (TmeA) SalI F | CC**GGTACC**ATGAGTATTCGACCTACTAATGGGAG | Ectopic expression |
| CT694 (TmeA) XhoI R | CC**CTCGAGTTA**GTCTAAGAAAACAGAAGAAGTT | Ectopic expression |
| CT711 KpnI F | CC**GGTACC**GTGTCAATACAACCTACATCCATTTC | Ectopic expression |
| CT711 XhoI R | CC**CTCGAGTTA**TTTAAATCTACGGATCAACTTAGCAA | Ectopic expression |
| CT712 KpnI F | CC**GGTACC**ATGAGAAACCATCCGATTCC | Ectopic expression |
| CT712 XbaI R | CC**TCTAGA**TTAGCTAGAAGCCAATGTTCTATATACATT | Ectopic expression |
| CT738 KpnI F | CC**GGTACC**ATGGAAGGTTTTTTCCCTATAGC | Ectopic expression |
| CT738 XhoI R | CC**CTCGAG**TACAAGGCTCGGAGCAGAA | Ectopic expression |
| CT848 KpnI F | CC**GGTACC**ATGTGGCATAAAGAACCAATGTATG | Ectopic expression |
| CT848 XhoI R | CC**CTCGAG**TTAGATATTCGCGATCAAGCTAACG | Ectopic expression |
| CT849 KpnI F | CC**GGTACC**ATGTCAGCACCAACCTCACA | Ectopic expression |
| CT849 XhoI R | CC**CTCGAG**TTAAGACAGGGGTTTATTTAATTGGTTAAC | Ectopic expression |
