# Supplementary material for: Global mapping of the Chlamydia trachomatis conventional secreted effector – host interactome reveals CebN interacts with nucleoporins and Rae1 to impede STAT1 nuclear translocation
Source: bioRxiv. 2024 Apr 25:2024.04.25.587017. Preprint. [Version 1] doi: 10.1101/2024.04.25.587017 (PMC11071493; doi:10.1101/2024.04.25.587017)
Supplement: Supplementary file 1 [file media-1.zip › Sup/Supp Materials FINAL.docx]

**SUPPLEMENTAL MATERIALS**

**Fig. S1.** Expression of CyaA and BlaM tagged effectors. Lysates from HeLa cells infected with *C.t.* strains expressing CyaA (A) or BlaM (B) tagged effector proteins were probed with anti-CyaA (A) or anti-BlaM (B) antibodies to confirm expression of the effector of interest. HSP was used as a *C.t.* loading control (A,B). 

**Fig. S2.** Molecular function and biological processes targeted by *C.t.* secreted effector proteins. (A, B) MiST was used to identify high confidence interacting partners for each effector screened. Those with a MiST score ≥0.69 were considered significant and used for further analysis of molecular function (A) and biological pathway (B) targeted by each effector. Size of the dot correlates with the number of unique prey(s) and color correlates with average MiST score for those prey.

**Fig. S3.** Visualization of STRING PPI analysis. (A-M) STRING and Cytoscape were used to generate STRING graphs to evaluate potential connections between prey with significant MiST scores (≥0.69). Only those effectors with prey that contained links were visualized. Darker, thicker lines correlate with more known and predicted interactions between node proteins.

**Fig. S4.** Proteolytic cleavage of NUP153 is an artifact of CPAF activity during sample processing. HeLa cells were left uninfected or infected with WT L2 or a CPAF mutant (*cpa::cat*). Lysates were used for immunoblotting with antibodies against NUP153.
